# Curving Tuberculosis: Current Trends and Future Needs

**DOI:** 10.5334/aogh.2415

**Published:** 2019-01-22

**Authors:** B. Rojano, J. A. Caminero, M. Hayek

**Affiliations:** 1 Division of General Internal Medicine, Department of Medicine, Icahn School of Medicine at Mount Sinai, New York, US; 2 University Hospital of Gran Canaria “Dr. Negrin,” Las Palmas, ES; 3 Multi Drug Resistant Tuberculosis Unit, International Union against Tuberculosis and Lung Disease (The Union), Paris, FR; 4 Department of Internal Medicine, Infectious Disease Unit, University Hospital of Tenerife “La Candelaria”, ES

## Abstract

Tuberculosis (TB) presents new challenges as a global public health problem, especially at a time of increasing threats to some particular patients due to Human Immunodeficiency Virus (HIV) infection and multidrug-resistant (MDR) and extensively drug-resistant (XDR) strains of Mycobacterium tuberculosis. The World Health Assembly strives to reduce TB deaths by 95% and to decrease TB incidence by 95% by 2035. However, new approaches are necessary in order to attain these objectives. Such approaches include active ascertainment of cases in high risk populations, increasing the availability of accurate point-of-care testing, rapid detection of drug resistance, novel vaccines, and new prophylaxis and treatment regimens (particularly for MDR and XDR TB). The ultimate objective of those programs is to develop highly effective drug regimens that can achieve high cure rates regardless of strains’ resistance patterns.

## Introduction: Global Burden of Tuberculosis

Mycobacterium Tuberculosis is an ancient and highly harmful successful human pathogen. Despite the advent of effective antimicrobial drugs, tuberculosis (TB) is still the most important infectious disease in humans and remains a leading cause of mortality worldwide [1,2].

In 1993, the World Health Organization (WHO) declared TB a global public health emergency [3]. Since then, TB incidence has fallen by an average of 1.5% per year and is now 18% lower than in 2000. However, the 2015 update of the WHO Global Tuberculosis Report [2] estimated that there were still approximately 10.4 million (5.9 million men, 3.5 million women, and 1 million children) incident cases worldwide and TB was considered the underlying cause of 1.8 million deaths, 400,000 of whom were among HIV-positive individuals [4].

TB is relatively simple and inexpensive to diagnose, in most cases can be cured with well-tolerated, effective, and low-cost treatments [5,6,7]. It has been estimated that 37 million patients were cured between 2000 and 2013, ascribed to advanced diagnostic methods in conjunction with effective treatments [2]. However, multidrug resistant tuberculosis (MDR-TB) remains a major challenge to achieving complete disease control.

The development of drug resistant TB strains is multifactorial. When TB bacteria replicate, some naturally mutate and become resistant to anti-TB drugs. TB treatment subsequent kills the non-mutated bacteria, leading to a selective survival of mutated, drug-resistant organisms. In 2015, it was estimated that approximately 580,000 individuals were carriers of rifampicin (RIF) and/or isoniazid (INH) and rifampicin-resistant (MDR) TB strains globally and that about 250,000 deaths were caused by those strains [2]. Moreover, only 25% of those cases were reported and just the 52% were successfully treated [2,8,9]. In 2006, extensively drug-resistant TB (XDR-TB; MDR plus resistance to any fluoroquinolone and, at least, one of the three second line injectable drug) also emerged as a more serious form of multidrug-resistant TB and a severe threat to public health, especially in countries with a high prevalence of HIV. Through 2015, XDR-TB cases have been reported in 105 countries and it is estimated 10% of cases of MDR-TB are XDR-TB [10,11].

## Diagnosing TB: Conventional Methods and Molecular Procedures

WHO’s global strategy for TB control prioritizes early diagnosis, which should include systematic screening of latent TB (contacts and high-risk groups) [12] and universal availability of drug susceptibility testing (DST). Unfortunately, of the 10.4 million TB cases estimated in 2015, more than 4 million remain undiagnosed and thus, continue to spread the disease [2]. Achieving global TB control requires that all of those cases be identified and effectively treated.

Nowadays, TB diagnosis is still based in many countries on clinical suspicion, radiography and microbiological tests, performed by microscopic examination of sputum, a 120-year-old method. Bacilloscopy based on sputum smears is rapid and inexpensive but has a moderate sensitivity (50–70%), even lower (30–60%) in HIV-infected patients [13,14]. Sputum culture is much more accurate for TB detection, but is expensive, time consuming and not available in some limited-resource settings.

Despite those challenges, the landscape for new TB diagnostics is promising as there is a robust pipeline of novel technologies including smaller, simpler and robust tests expected to become available in the near future [15]. Research is aimed to deliver results in hours, including DST assessments; leading to decrease time to treatment, enabling point-of-care testing programs [15], which are required to confirm the presence of RR/MDR/XDR-TB and select the most appropriate combination of drugs. New technologies include.

### Latent Infection

Latent tuberculosis infection (LTBI) is a state of persistent immune response to stimulation by *Mycobacterium Tuberculosis* antigens without evidence of active TB. Tools for reliable identification of patients with LTBI are required for a rational use of preventive therapy. Nowadays, the most applied diagnostic tools for LTBI are the in vivo Tuberculin Skin Test (TST) and the ex vivo Interferon-γ Release Assays (IGRAs). While useful, these tests are unable to determine the likelihood of progressing to active TB.

TST reveals the body’s hypersensitivity to the presence of TB bacillus proteins, a state acquired in most cases after initial infection. The assay consists of an intradermal injection of 0.1 ml of tuberculin purified protein derivative (PPD) into the inner surface of the forearm. Reading should be made 48–96 hours later by measuring the skin induration caused by the reaction. Although very simple, this test requires standardization, training and appropriate supervision. Bacillus Calmette-Guerin (BGC) vaccination can also cause hypersensitivity, leading to false positive results. Conversely, IGRAs detect circulating T-cells responsive to specific Mycobacterium Tuberculosis antigens, which are absent after BCG vaccination as well as after exposure to most non-tuberculosis Mycobacteria [16]. However, a recent meta-analysis comparing the accuracy of IGRAs vs. TST for the diagnosis of LTBI concluded that IGRAs may be better only in immune competent children aged >5 years residing in high-income areas. Even in these subjects, IGRAs sensitivity was only 67–86%, indicating that neither test may rule out LTBI nor confirm with certainty a true negative diagnosis [17]. The IGRAS currently in use primarily elicit a CD4+ response, but evidence supports the important contribution of CD8+ T-cells in host defense against TB. A new generation of QFT-GIT, the QuantiFERON-TB Gold Plus (QFT-Plus), contains peptides able to stimulate IFN-γ production by both CD4+ and CD8+ T-cells. Early data suggest that this test offers higher sensitivity and specificity, particularly in patients at the highest risk for TB-infection and in immunocompromised patients [18,19]. However, larger studies are needed to confirm the accuracy of the QFT Plus [20].

Per current WHO recommendations [21,22] and other international guidelines [23,24] either TST or IGRAs can be used to identify candidates for LTBI treatment in high and middle-income countries. The TST is more affordable and is therefore preferred in low-income regions, even if is less specific than IGRAs in vaccinated patients [17]. TST should be also used in children, patients with immunodeficiency, people who live with patients infected with TB and health care workers [25,26].

### Active Tuberculosis

TB diagnosis is still based on the observance of clinical symptoms (unexplained weight loss, decreased appetite, night sweats, fever, fatigue, coughing for >2 weeks, hemoptysis, chest pain, etc.); radiographic findings; and microbiological testing. Chest X-ray is the most sensitive (>95%) test for the diagnosis of pulmonary TB in immune-competent patients and, for this reason, is probably the best strategy for screening for TB.

Bacteriological procedures are the most specific and the only accepted diagnostic test to confirm a diagnosis of TB. These include:

1. Bacilloscopy: microscopic assessment of sputum for *Mycobacterium* TB is the initially recommended test due to its low cost and simplicity. However, the role of Bacilloscopy is limited due to moderate sensitivity (60–70%) and possible false positives due to identification of atypical Mycobacteria (specificity 95–98%) [5].
2. Culture: sputum culture (especially liquid media) is the most sensitive bacteriological method available and considered the diagnostic gold standard.
3. Mycobateria specie identification, necessary to confirm TB vs infection by other Mycobacteria.
4. Drug susceptibility test or Antibiogram: in vitro testing of anti-TB drug sensitivity is critical for selecting an appropriate TB regimen but takes several weeks. Novel molecular methods that can provide results in hours have recently become available.

### Drug-resistant TB

The immediate future for the management of TB will involve the use of rapid molecular microbiological techniques [27]. Susceptibility drug tests can be either phenotypic or genotypic. Phenotypic DST, widely used in developing countries, is based on assessing bacterial growth with fixed concentrations of first (e.g., INH, RIF, ethambutol [EMB], and streptomycin [SM]) or secondary-line anti-TB drugs. This method is simple and cost-effective; however, it leads to considerable delays and is unreliable for EMB and other drugs. Genotypic methods take advantage of specific mutations that are associated with response to particular drugs [28]. Molecular methods can be performed directly on sputum or other samples, and can provide results (including drug susceptibility) in 24 to 48 hours. The most used methods are:

1. GenXpert Mycobacterium Tuberculosis/RIFAMPICIN (GENXpert MTB/RIF), is a nucleic acid amplification assay conducted in sputum that simultaneously tests for presence of Mycobacterium Tuberculosis DNA and RIF resistance. This is a simple and reproducible test with a 90% sensitivity (reaching 98% in patients with positive bacilloscopy but dropping to 70% in smear negative disease) and 99% specificity. The assay is automated and does not require laboratory infrastructure. The identification of RIF resistance is often considered a marker of MDR-TB (in 95% of cases) with INH resistance [29]. WHO has recently recommended Gene-Xpert as an initial diagnosis test for patients with HIV suspected of having TB or for patients who are considered at risk for RIF resistance or MDR-TB. Similar assays currently under development include: the GeneXpert Omni, also intended for TB testing combined with RIF resistance assessment but uses a cheaper and more portable platform, potentially allowing for bedside testing and the Xpert MTB/RIF Ultra, a more sensitive and specific assessment which may improve detection of TB in smear-negative patients.
2. GenoType, also known as line probe assay (LPA), a test that can detect RIF or INH resistance within 48 hours [29,30]. Additionally, the test is very accurate for detecting resistance to fluoroquinolones and second line injectable [31]. The assay is based on a conventional polymerase chain reaction, thus, requires access to a biomolecular laboratory.

## Treatment: Current Regimens with Emerging Drugs

Although current regimens for drug-sensitive TB (2 months of 4 drugs, 4 months of 2 drugs) are highly effective under trial conditions [6,7], cure rates drop to approximately 85% when used in real life [32]. WHO-recommended regimens for drug-sensitive [33] and drug-resistant TB [34] present several challenges including a long duration, although there have been recent improvements with the introduction of shorter MDR-TB regimens.

Patients with drug-resistant TB must resort to second-line drugs, which are less effective and require longer, more toxic and costly treatment courses. These factors lead to reduced patient adherence and lower success rates. Indeed, WHO reported that, on average, only 22 to 26% of patients with XDR-TB successfully complete treatment [35], and those rates only increased to 52% in the cases with MDR-TB. Treatment is based on a combination of drugs to prevent emergence of resistance over an extended period of time to ensure cure and prevent relapses (i.e., the re-emergence of clinical symptoms after initially effective anti-TB treatment).

### Classification of TB Drugs (Table 1)

The current classification divides anti-TB drugs in 4 groups: A, B, C, D [36]. This classification is specifically designed to incorporate the treatment of RIF-resistant or MDR-TB cases [37].

– Group A includes fluoroquinolones (high dose of Levofloxacin, Moxifloxacin, and Gatifloxacin). These are considered core drugs due to their bactericidal and sterilizing activity, as well as good safety profile.
– Group B includes injectable drugs (Streptomycin, Amikacin, Kanamycin and Capreomycin), which have quality bactericidal activity but poorer safety profile than group A medications.
– Group C is composed by Ethionamide, Prothionamide, Cycloserine/Terizidone, Clofazimine and Linezolid. These drugs are recommended as core second-line medicines for MDR-TB given growing evidence about their efficacy and tolerability.
– Group D includes non-core MDR-TB drugs and is divided in 3 subgroups: D1: high dose INH, EMB, Pirazinamide; D2: Bedaquiline and Delamanid; and D3: Meropenem, Para-Aminosalicylic Acid, Imipenem-Cilastatin, Amoxicillin-Clavulanate, and Clarithromycin.

**Table 1:** World Health Organization Classification of Tuberculosis Drugs.

Group	Drugs

A: Fluoroquinolones	Levofloxacin, Moxifloxacin, Gatifloxacin
B: Second line injectable drugs	Streptomycin, Amikacin, Kanamycin, Capreomycin
C: Other core second-line drugs	Ethionamide, Prothionamide, Cycloserine/Terizidone, Clofazimine, Linezolid
D: Non-core MDR-TB drugs	High dose Isoniazid, Ethambutol, Pyrazinamide Bedaquiline, Delamanid Meropenem, Para-Aminosalicylic Acid, Imipenem-Cilastatin, Amoxicillin-Clavulanate, Clarithromycin

### New trends in TB

After 50 years of minimal anti-TB drug development, a promising pipeline has recently emerged through the repurposing of old drugs and discovery of new compounds. Combinations of new and existing drugs are being assessed to shorten the duration of therapy and to effectively treat cases of MDR TB. There has also been progress in development of drugs that are active against dormant populations of Mycobacterium tuberculosis.

Bedaquiline is the first new anti-TB drug approved by the Food and Drug Administration (FDA) in 50 years to be used as part of combination treatment with MDR-TB for adults when other alternatives are not available [38]. It targets both active and dormant bacilli [39,40], and therefore has the profile required of a core drug. A randomized phase II trial reported faster sputum-culture negative conversion in the patients receiving Bedaquiline (hazard ratio: 11.8, 95% confidence interval: 2.3–61.3) [41]. Cure rates were 58% for patients who received Bedaquiline compared to 32% for controls (p = 0.003) [42]. However, another trial showed a small increase in all-cause mortality among patients in the Bedaquiline arm [43]. Common adverse reactions associated with Bedaquiline were an increased QTc interval and cross-resistance with Clofazimine [44,45].

Delamanid, approved by the European Medicine Agency, is a metronidazole derivate with bactericidal and sterilizing activity [46,47]. Unlike Bedaquiline, Delamanid does not show cross-resistance with other anti-TB drugs. A recent study showed that when added to standard-of-care chemotherapy in patients with MDR TB, Delamanid was associated with a significantly higher proportion of sputum culture conversion at two months of treatment initiation (45% versus 29%) [48,49]. QTc prolongation was also observed in Delamanid-treated patients [50,51].

Linezolid may be also considered an “essential” drug. Two small randomized clinical trials [52,53] and various meta-analyses [54,55,50] have shown its effectiveness for treating MDR-TB, especially XDR-TB. However, there are two downsides of Linezolid: its cost and toxicity profile (hematological disorders and polyneuropathies) when administered for more than 6–8 weeks [56]. Sutezolid is a Linezolid analogue which has shown greater anti-mycobacterial activity in clinical trials and is active against non-replicating Mycobacterium Tuberculosis both in vitro and in vivo [57]. Recent studies showed highly additive effects for combination of this drug with rifamycins [58]. In addition, phase I trials showed sputum early bactericidal activity in patients with TB and no hematological toxic effects [59].

### Regimens for new cases of TB

WHO currently recommends a 6-month treatment regimen for TB; combining four effective medications, three of which should be “essential” drugs (INH, RIF, Z), two with good bactericidal activity (INH, RIF), and another two with good sterilizing capacity (RIF, Z). EMB has the role of an “accompanying” drug; supporting the core drugs to avoid resistance selection. EMB is not included in the regimen to eliminate bacilli and, in theory, it is no longer necessary after the bacteriological conversion is achieved [60]. Thus, patients are treated with INH, RIF, pyrazinamide and EMB for the first two months (intensive phase), followed by INH and RIF for the remaining 4 months (continuation phase) [34]. Importantly, if a core drug cannot be used because of documented resistance or toxicity, it should be replaced by another drug with a similar efficacy (i.e., bactericidal and sterilizing properties). Similarly, an accompanying drug should be replaced by another drug with an equivalent action. Patients with HIV infection and those with certain forms of extrapulmonary TB (meningeal, disseminated) may have their treatment extended to 9–12 months [66, 61].

### WHO Recommendations for Drug Resistant TB

Following the current WHO recommendations, supported by a multi-center study of 1,200 patients, a stepwise approach using one of the newer shorter regimens is recommended for MDR-TB [62]. This approach is likely to improve safety, tolerability and adherence, improving the patients’ outcomes [67]. The specific regimen may be chosen on a standardized or empirical basis and then switched to individualized therapy after results of DST become available [36]. Drugs used in the treatment of MDR and XDR-TB are less efficacious and less well tolerated when used for prolonged periods, leading to a high risk of toxic effects. The treatment in these cases requires combination regimens.

– **MDR-TB cases not resistant to and/or never treated with second-line anti-TB drugs:** WHO recommends a new short regimen consisting of 4–6 months of kanamycin, high dose Koxifloxacin, Prothionamide, Clofazimine, Pyrazinamide, high dose INH and EMB followed by five months of high dose Moxifloxacin, Clofazimine, Pyrazinamide and EMB. This shorter regimen (9–12 months) has produced excellent outcomes in recent studies leading to relapse-free success in near 90% of patients treated, [63,64] much higher than the global success of the conventional MDR-TB regimen (52%). The strongest risk factor for an unfavorable outcome is a high level of resistance to fluoroquinolone, particularly when compounded by initial Pyrazinamide resistance [71].
– **Multidrug resistance TB or rifampicin-resistant:** MDR-TB treatment is recommended for all patients with RIF-resistant tuberculosis, regardless of whether isoniazid resistance is confirmed.
– When the shorter regimens are not applicable, treatment should include at least five effective drugs during the intensive period. These drugs may include Pyrazinamide and four second line core drugs (one from group A; one from group B; and at least two from group C). If this regimen cannot be followed due to problems with tolerance, contraindication or drug availability, then the total of five drugs must be complemented by a group D2 or D3 drug. This regimen must be strengthened with high-dose INH and/or EMB.
– If INH resistance is absent or unknown + RIF resistance, all patients (child or adult) must follow the previous regimens to which INH is added, but it should not be counted among the four active drugs.
– Surgical interventions in patients with MDR-TB: elective partial lung resection (lobectomy or wedge resection) may be used alongside a recommended MDR-TB regimen.

## Conclusion

Substantial progress has been made in the early diagnosis of TB, DST and in the development of new anti-TB regimens throughout the past decade. New molecular tests have made rapid diagnosis of active disease possible, leading to an estimated 37 million lives saved worldwide between 2000 and 2013. However, global TB control remains suboptimal. Future efforts should focus on the implementation of newly developed regimens as well as continuous research to identify shorter, better-tolerated anti-TB treatments. Additionally, there is a need for better biomarkers and newer drugs to achieve improved outcomes in patients with drug-resistant TB and/or HIV co-infection.
